# Access to treatment before and after Medicare coverage of opioid treatment programs

**DOI:** 10.1093/haschl/qxae076

**Published:** 2024-06-06

**Authors:** Ruijie Liu, Tamara Beetham, Helen Newton, Susan H Busch

**Affiliations:** Department of Health Policy and Management, Yale School of Public Health, New Haven, CT 06510, United States; Department of Health Policy and Management, Yale School of Public Health, New Haven, CT 06510, United States; Department of Family Medicine, University of North Carolina School of Medicine, Chapel Hill, NC 27599, United States; Department of Health Policy and Management, Yale School of Public Health, New Haven, CT 06510, United States

**Keywords:** Medicare, opioid treatment program (OTP), opioid use disorder (OUD), methadone, for-profit facilities, racial differences, access to care, health policy

## Abstract

Since January 2020, Medicare has covered opioid use disorder (OUD) treatment services at opioid treatment programs (OTPs), the only outpatient settings allowed to dispense methadone for treating OUD. This study examined policy-associated changes in Medicare acceptance and the availability of four OUD treatment services (ongoing buprenorphine, HIV/AIDS education, employment services, and comprehensive mental health assessment), by for-profit status, and county-level changes in Medicare-accepting-OTPs access, by sociodemographic characteristics (racial composition, poverty rate, and rurality). Using data from the 2019-2022 National Directory of Drug and Alcohol Abuse Treatment Facilities, we found Medicare acceptance increased from 21.31% in 2018 to 80.76% in 2021. The availability of the four treatment services increased, but no increases were significantly associated with Medicare coverage. While county-level OTP access significantly improved, counties with higher rates of non-White residents experienced an additional average increase of 0.86 Medicare-accepting-OTPs (95% CI, 0.05–1.67) compared to those without higher rates of non-White populations. Overall, Medicare coverage was associated with improved OTP access, not ancillary services.

## Teaser

Opioid treatment programs (OTPs) are the only outpatient settings allowed to dispense methadone as a medication for treating opioid use disorder (OUD). However, Medicare fee-for-service did not cover treatment services for OUD furnished in OTPs until 2020. Adding to previous literature, we examined changes in the availability of OUD treatment services at OTPs and in county-level access to Medicare-accepting-OTPs associated with the nationwide Medicare coverage expansion. Our findings highlight the role of for-profit facilities and facilities in non-White communities in expanding access to OUD treatment among Medicare population. This study helps better understand policy-associated improvements in the treatment access and suggests potential policy strategies to address the ongoing opioid epidemic.

## Introduction

In 2021, 5.6 million people in the United States had opioid use disorder (OUD).^1^ About 190 people per day died from opioid-involved drug overdoses in 2020, a 38% increase from 2019.^2^ Opioid treatment programs (OTPs) remain the sole outpatient settings allowed to dispense methadone for treating OUD (US pharmacies can dispense methadone for pain management), although recent policy efforts have proposed expanding access to other community settings.^3,4^ Given the rising prevalence of synthetic high-dose opioids such as fentanyl, access to methadone may be especially important as prior studies indicate methadone has better or equal effectiveness than buprenorphine when treating patients using fentanyl.^5-8^ Opioid treatment programs are often the only outpatient facilities where patients can access all three medications for OUD (MOUD): methadone, buprenorphine, and naltrexone.^9^ Racial minorities, especially Black patients, are disproportionately burdened by opioid overdose fatalities relative to White patients yet less likely to have access to any MOUD.^10-12^ Moreover, among patients treated with MOUD, Black patients are more likely to be treated at OTPs. Expanding access to treatment at OTPs has the potential to reduce racial disparities in any MOUD receipt if it improves access to MOUD overall, yet if methadone has more punitive aspects than other MOUD, it may further stigmatize treatment among this patient group.^13,14^

Despite the importance of OTPs, Medicare fee-for-service (FFS) did not cover methadone treatment or services for OUD in OTPs prior to 2020, resulting in restricted access to OUD treatment among Medicare beneficiaries. In 2019, only 10.5% of Medicare-covered OUD patients reported past-year MOUD uptake, compared to 45.1% of Medicaid-covered OUD patients and 24.3% of patients with private insurance.^15^ In addition to MOUD, federal law requires OTPs to make ancillary OUD treatment services available, including vocational, educational, and other assessment and treatment services.^16^ The national practice guideline for OUD treatment developed by the American Society of Addiction Medicine (ASAM) notes the benefits of offering ancillary services, determined through shared decision making, in conjunction with MOUD.^17^ For example, the ASAM recommends screening patients’ mental health status for psychiatric disorders given the high prevalence of co-occurring mental health conditions among patients with OUD. Although MOUD is effective, patients may seek ancillary services tailored to their individual needs, and evidence demonstrates that offering these services with MOUD can reduce illicit opioid use and infectious disease risks, increase treatment retention, improve social functioning, and serve as a factor in achieving sustained recovery.^18^ While ancillary supportive services should never be a requirement for MOUD, availability of services offered by OTPs is consistent with best practice guidelines, indicating that all patients should have the option to utilize these services through a shared decision-making process.^17,19^

Effective January 1, 2020, the Substance Use-Disorder Prevention that Promotes Opioid Recovery and Treatment for Patients and Communities Act established Medicare benefits for OUD treatment services delivered at OTPs.^20^ Medicare FFS began covering OTP services with a bundled payment for a weekly episode of care, making methadone available to its beneficiaries as an OUD treatment option for the first time. Recent studies suggested this policy improved county-level methadone availability and increased methadone dispensing rates, mainly driven by Medicare beneficiaries with Medicaid benefits (dual eligibles).^21,22^ Medicare generally provided higher reimbursements than Medicaid: The national average Medicaid bundled payment was only 56% of the Medicare payment for methadone treatment and 64% for ancillary services at OTPs in 2021.^23^ Consequently, this new Medicare benefit may increase revenue for many OTPs treating dual eligibles and these additional resources might be used to newly offer ASAM-recommended services, particularly among mission-driven OTPs. Bundled payments that include ancillary services could also encourage offering a wider array of services. Given that regulations and guidelines emphasize these ancillary services be offered, any enhanced federal oversight that accompanied Medicare coverage may encourage service availability.

However, there could be differential effects by OTP ownership status. For-profit facilities may minimize their costs by stinting on ancillary services under the bundled payment system. Previous research suggests that for-profit substance use disorder (SUD) treatment facilities were more responsive to changes in health insurance policy, primarily driven by their financial incentives, than nonprofit facilities.^24^ Moreover, recent literature indicated that private equity-owned healthcare facilities were associated with poorer health outcomes.^25,26^ Though the new Medicare coverage and bundled payments may affect service offerings, changes in the availability of OUD treatment services have not been studied.

In this study, we used a national panel of OTPs operating from 2018 to 2021 to assess changes in whether OTPs accepted Medicare insurance and the availability of OUD treatment services at OTPs before (2018 and 2019) and after (2020 and 2021) the Medicare coverage expansion. As for-profit OTPs have become dominant in the past decade, we further analyzed differential changes in Medicare acceptance and service availability between for-profit and nonprofit OTPs.^27^ Additionally, we examined changes in county-level access to OTPs for Medicare beneficiaries by county racial composition, poverty rates, and rurality, to elucidate any changes in the role of OTPs in increasing access to MOUD for historically under-resourced communities.

## Data and methods

### Data

We used data from the 2019-2022 National Directory of Drug and Alcohol Abuse Treatment Facilities to identify OTPs.^27^ These national directories include SUD treatment specialty facilities that are state-approved or certified for inclusion and provide comprehensive self-reported facility information including address, ownership, accepted payments, and available treatment services. Facilities listed in the 2019-2021 directories responded to the 2018-2020 National Survey of Substance Abuse Treatment Services (N-SSATS). After 2021, the Substance Abuse and Mental Health Services Administration (SAMHSA) combined the N-SSATS and the National Mental Health Services Survey to create the National Substance Use and Mental Health Services Survey (N-SUMHSS). Thus, those listed in the 2022 directory are facilities that responded to the 2021 N-SUMHSS. The N-SSATS is an annual census of SUD treatment specialty facilities conducted by the SAMHSA, with response rates over 90%. The response rate to the 2021 N-SUMHSS was 78.8%. We also obtained supplementary data on county-level Medicare enrollment and sociodemographic characteristics from the Centers for Medicare & Medicaid Services and the American Community Survey.^28,29^

### Study sample

Using facility name, ZIP code, and facility-reported OTP status, we identified 2246 unique OTPs (6030 OTP–year observations) for surveys collected between 2018 and 2021. We constructed a facility-level balanced panel omitting OTPs for which we did not have four years of data, resulting in 868 OTPs (3472 OTP–year observations) for analyses. The difference in sample size was likely attributable to facility closures and openings, given the high response rates to the annual survey and that over 95% of these responded OTPs were listed in the national directories.^27^

For county-level analyses, we began by including all counties in the United States (3293 in 2018 and 3281 in 2021) to capture aggregate changes. Next, we created a balanced panel of 778 counties (3112 county–year observations) that experienced changes in access to OTPs accepting Medicare (Medicare-accepting-OTPs) during the study period to examine heterogeneity.

### Outcomes

Facility-level outcomes included whether OTPs reported accepting Medicare payment and offering pharmacotherapy and ancillary services for OUD treatment. Because OTPs universally reported offering methadone, we chose to focus on the availability of other services that are markers of high-quality OUD treatment. Specifically, we considered whether OTPs offered ongoing buprenorphine, HIV/AIDS education and support, employment services, and comprehensive mental health assessment. These services are recommended by the ASAM and the SAMHSA to reduce the risk of co-occurring disorders, such as mental illness and HIV, and to enhance treatment retention.^9,17,30,31^ County-level outcomes included the presence and the number of Medicare-accepting-OTPs within counties.

### Independent variables

In this descriptive study, year indicators representing the implementation of the Medicare coverage expansion policy were the key independent variables used to examine facility-level and county-level changes. Additionally, we constructed indicators of whether an OTP was reported as for-profit, whether the county was in the top quartile for percentage of non-White residents (more than 27.02%) in 2018, whether the county had more than 10% of residents living below the federal poverty line in 2018, and whether the county was rural, defined as not being part of a metropolitan statistical area.

### Statistical analyses

We estimated linear regression models using the balanced panel of OTPs with facility-fixed effects to describe changes in Medicare acceptance and service availability at OTPs before and after the 2020 Medicare coverage expansion. This approach allowed us to control for time-invariant OTP characteristics and look at changes within facilities over time. For Medicare acceptance, our outcome variable was an indicator of whether the OTP accepted Medicare in a given year. We then assessed the differential changes by ownership status by examining the interaction terms between for-profit facility status and year variables in some models. Similar analyses were conducted to describe the aggregate and heterogeneous changes in the availability of each of the four OUD treatment services.

We examined changes in county-level OTP access for Medicare beneficiaries by estimating linear regression models with county-fixed effects among counties that experienced changes in the number of Medicare-accepting-OTPs. In these analyses, we used county as our unit of analysis rather than facility to determine whether counties’ racial composition, poverty rates, and rurality were associated with county-level changes in OTP access. We incorporated interaction terms between county characteristics and year variables and included state-fixed effects to control for states’ time-invariant characteristics. We employed Wald’s tests for all the coefficients of interest to examine the magnitude of annual changes.

We performed robustness checks to assess whether the results were robust to our choice of sample selection, estimation model, and other assumptions. First, for the OTP-level analyses, we created a sample with 2425 observations from 1341 OTPs that were in the full unbalanced panel and had consistent ownership status but were excluded from the balanced panel. We re-estimated changes in Medicare acceptance and service availability among these excluded OTPs to address sample selection concerns. To mitigate potential concerns of collinearity when assessing differential changes in county-level access, we conducted three regressions separately estimating the effects of racial composition, poverty, and rurality. Additionally, we performed sensitivity analyses for the heterogeneity of changes in service availability by OTPs’ Medicare acceptance status. Further details of the statistical analyses are given in the Appendix.

## Results

### Baseline characteristics of OTPs

In 2018, 185 (21.31%) of OTPs in the balanced panel reported accepting Medicare payment (Table 1). The majority (557 [64.17%]) of these OTPs were for-profit, with a lower Medicare acceptance rate (65 [11.67%]). Most OTPs reported offering ongoing buprenorphine (602 [69.35%]) and HIV/AIDS education and support services (709 [81.68%]), while the availability of employment services (401 [46.20%]) and comprehensive mental health assessment services (231 [26.61%]) was limited. Nonprofit OTPs were more likely to offer ancillary services compared to for-profit OTPs, whereas for-profit OTPs were more likely to offer ongoing buprenorphine services.

**Table 1.** Baseline characteristics of OTPs in the balanced panel (2018).

	Balanced sample, no. (%)^a^			OTPs excluded due to ownership switch, no. (%)
Characteristic	Full analytic sample	For-profit OTPs	Nonprofit OTPs	
Total OTPs, no.	868	557	311	26
For-profit	557 (64.17)	557 (100.00)	0 (0.00)	NA
Accepting Medicare^b^	185 (21.31)	65 (11.67)	120 (38.59)	6 (23.08)
Treatment services				
Ongoing buprenorphine	602 (69.35)	396 (71.10)	206 (66.24)	20 (76.92)
HIV or AIDS education and support	709 (81.68)	433 (77.74)	276 (88.75)	15 (57.69)
Employment services	401 (46.20)	246 (44.17)	155 (49.84)	8 (30.77)
Comprehensive mental health assessment	231 (26.61)	62 (11.13)	169 (54.34)	1 (3.85)
County characteristics				
Rural	92 (10.60)	76 (13.64)	16 (5.14)	3 (11.54)
Top non-White population quartile^c^	364 (41.92)	209 (37.52)	155 (49.84)	17 (65.38)
> 10% below FPL^d^	653 (75.22)	426 (76.41)	232 (74.51)	21 (82.61)
Region				
Northeast	213 (24.54)	91 (16.34)	123 (39.41)	5 (20.00)
Midwest	138 (15.86)	75 (13.46)	63 (20.20)	2 (8.00)
South	320 (36.92)	260 (46.68)	60 (19.22)	9 (36.00)
West	197 (22.69)	131 (23.52)	66 (21.17)	9 (36.00)

OTP, opioid treatment program; FPL, federal poverty level.

Source: Authors’ analysis of the 2019 National Directory of Drug and Alcohol Abuse Treatment Facilities data.

^a^The full analytic sample of the balanced panel included 868 of the 894 OTPs listed in every national directory from 2019 to 2022, with 26 excluded due to changes in ownership.

^b^OTPs that reported accepting Medicare insurance. This may include Medicare FFS payments for services not related to opioid use disorder treatment and Medicare Advantage payments for treatment services related to opioid use disorder.

^c^Counties with more than 27.02% non-White residents in 2018.

^d^Counties with more than 10% residents living below the federal poverty line in 2018.

### Facility-level Medicare acceptance

**Figure 1.**
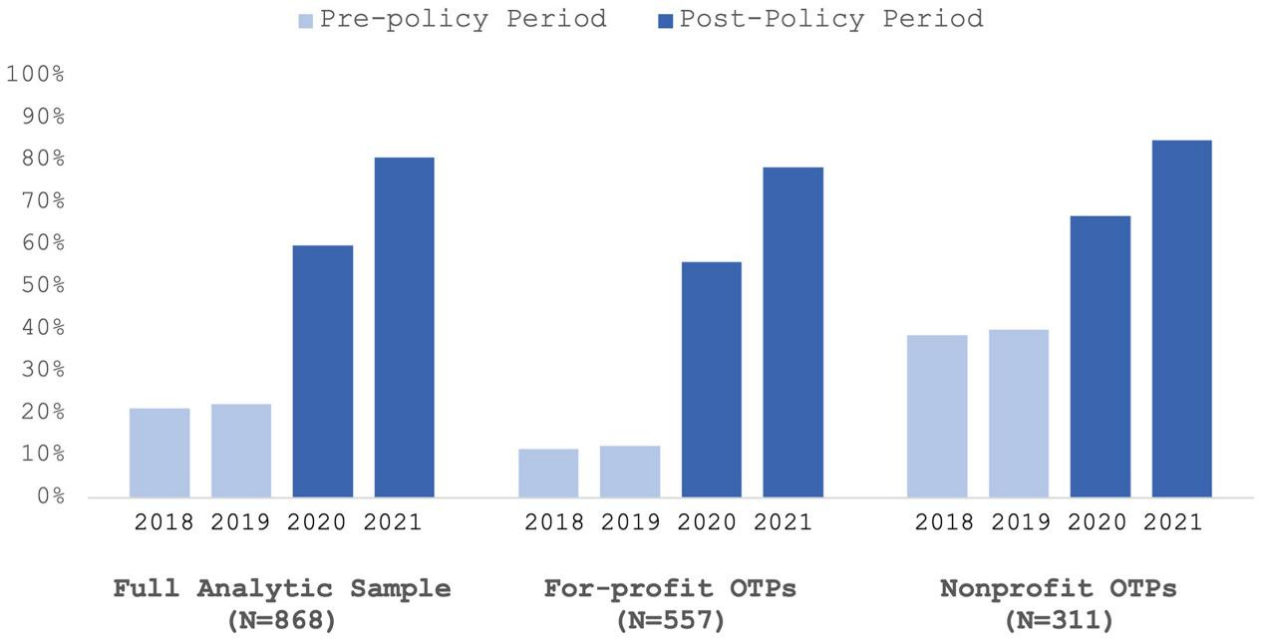
Percentage of OTPs accepting Medicare by ownership (2018-2021). Source: Authors’ analysis of National Directory of Drug and Alcohol Abuse Treatment Facilities data (2019-2022). Notes: Details on the number and percentage of OTPs in the balanced panel accepting Medicare are given in Appendix Table S1. Regression analyses results from the fixed effects model showed significant increases in Medicare acceptance rates in the analytic sample, nonprofit OTPs, and for-profit OTPs following the nationwide Medicare coverage expansion. Particularly, the increases in the for-profit OTPs were significantly higher compared to nonprofit OTPs. Wald test results indicated that the increases in Medicare acceptance from 2018 to 2019, in the full analytic sample, nonprofit OTPs, and for-profit OTPs, were significantly lower compared to the increases observed from 2019 to 2020 and 2020 to 2021. The full results of regression analyses and the Wald test are given in Appendix Table S2. OTP, opioid treatment program.

From 2018 to 2021, the share of Medicare-accepting-OTPs increased from 21.31% (185/868) to 80.76% (701/868), which was more pronounced among for-profit OTPs (from 11.67% [65/557] to 78.46% [437/557]) than among nonprofit OTPs (from 38.59% [120/311] to 84.89% [264/311]) (Figure 1 and Appendix Table S1). Consequently, although nonprofit OTPs had historically been more likely to accept Medicare payments, Medicare acceptance rates were comparable between for-profit and nonprofit OTPs in 2021. Regression analyses corroborate these findings (Appendix Table S2).

### OUD services availability at OTPs

**Figure 2.**
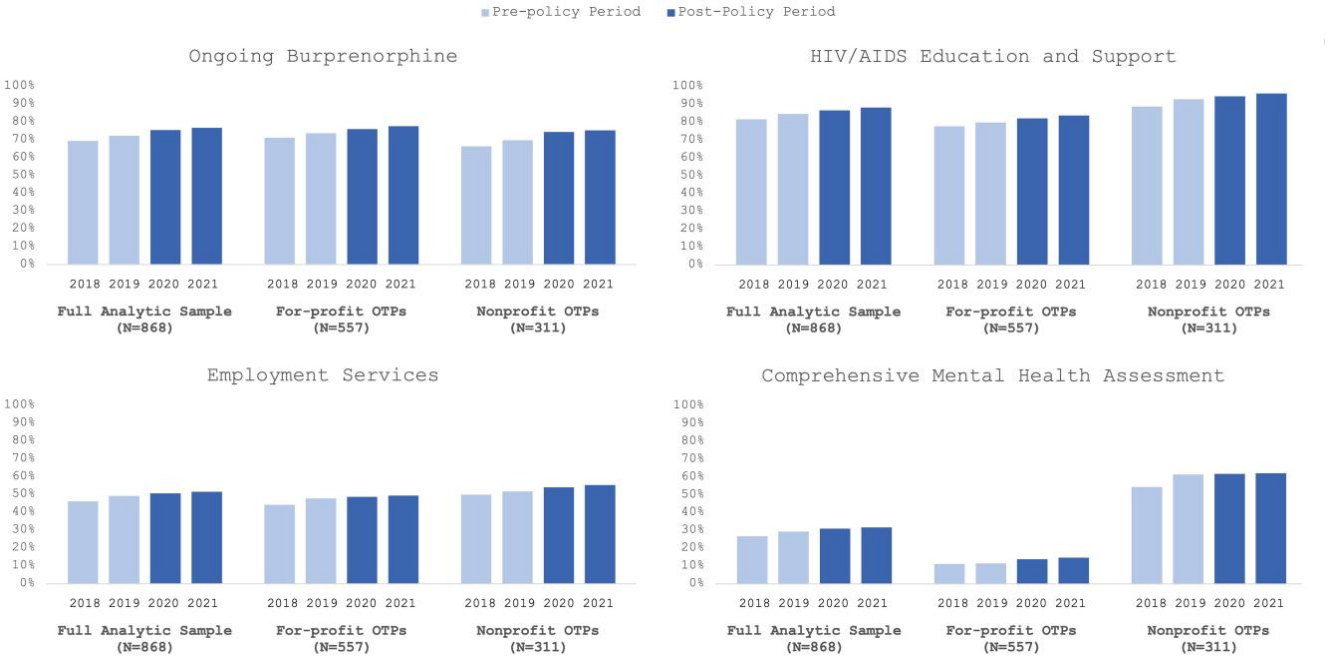
Percentage of OTPs offering OUD treatment services by ownership (2018-2021). Source: Authors’ analysis of National Directory of Drug and Alcohol Abuse Treatment Facilities data (2019-2022). Notes: Details on the number and percentage of OTPs in the balanced panel offering each OUD treatment service are given in Appendix Table S3. Regression analyses results from the fixed effects model showed significant temporal increases in the likelihood of OTPs offering each of the four OUD treatment services, but these increases were not associated with the Medicare coverage expansion. The full results of regression analyses and the Wald test are given in Appendix Table S4. OTP, opioid treatment program; OUD, opioid use disorder.

The availability of all four OUD services grew over the study period regardless of ownership, yet comprehensive mental health assessment services remained limited, available in only 31.68% (275/868) of the overall OTPs and in 14.72% (82/557) of the for-profit facilities in 2021 (Figure 2 and Appendix Table S3). Regression analyses suggest that improvements in service availability represented temporal trends and were not associated with the Medicare coverage expansion (Appendix Table S4).

### County-level access to OTPs

We observed an improvement in county-level access to OTPs for Medicare beneficiaries from 2018 to 2021. Among all counties in the United States, 17.92% (588/3281) had at least one Medicare-accepting-OTP in 2021, which had tripled from 5.01% (165/3293) in 2018 (Appendix Table S5).

**Figure 3.**
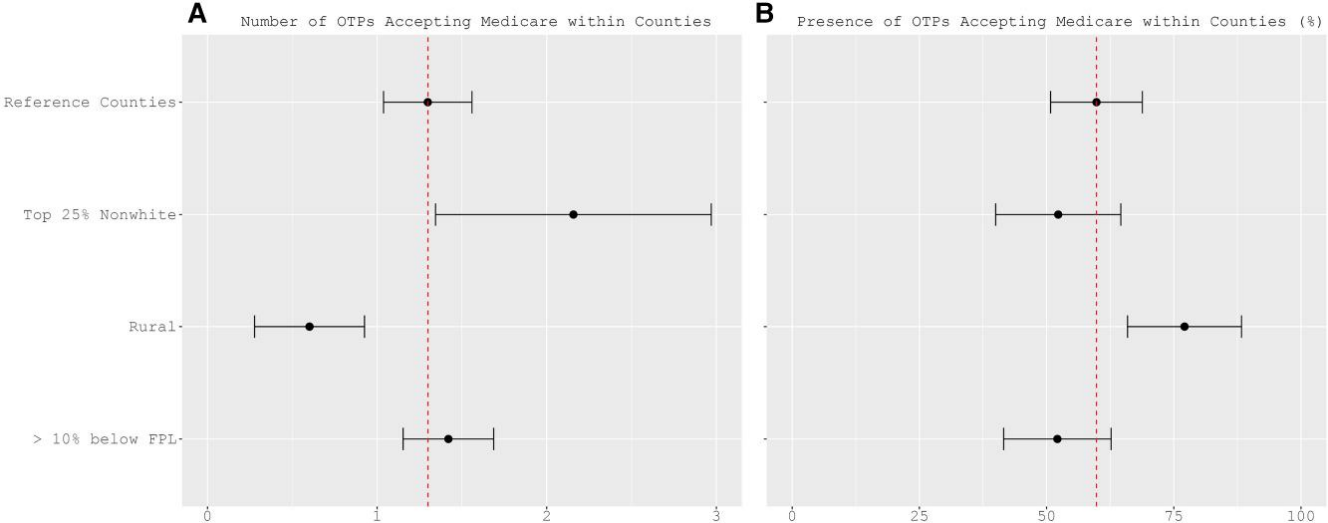
Adjusted differential changes in county-level access to OTPs for Medicare beneficiaries (2018-2021). Source: Authors’ analysis of National Directory of Drug and Alcohol Abuse Treatment Facilities data (2019-2022). Notes: A and B show the coefficients representing the average marginal effects on the number and presence of Medicare-accepting-OTPs within counties in 2021, compared to 2018, respectively. Reference counties were urban counties with a non-White resident percentage in the first to third quartile (<27.02%) and <10% of residents living below the federal poverty line in 2018. The regression analyses considered the 778 counties that experienced changes in access to Medicare-accepting-OTPs and used 2400 county–year observations from 600 counties. The other 178 counties were omitted due to missing information on county socioeconomic characteristics. The full results of regression analyses and the Wald test are given in Appendix Table S6. OTP, opioid treatment program; FPL, federal poverty level.

Within counties that saw changes in county-level access to Medicare-accepting-OTPs, regression analyses estimated the increases in the number and the presence of Medicare-accepting-OTPs. Reinforcing existing racial patterns related to access to methadone treatment, counties with the top quartile of non-White residents experienced a significantly higher increase in the number of Medicare-accepting-OTPs in 2021, with an increase of almost one additional Medicare-accepting-OTP than other counties (β: 0.86, 95% CI, 0.05–1.67) (Figure 3A and Appendix Table S6). Rural counties were 17 percentage points more likely to have a change from no Medicare-accepting-OTPs to at least one Medicare-accepting-OTP in 2021 than other counties (Figure 3B, β: 0.17, 95% CI, 0.06–0.29). However, they saw a smaller growth in the number of such OTPs (β: −0.70, 95% CI, −1.01 to −0.38) compared to other counties.

All findings from robustness checks aligned with our primary analyses. Appendix Tables S7 and S8 show the baseline characteristics and annual Medicare acceptance and service availability of the alternative sample, respectively. The full results of the regression analyses are provided in Appendix Tables S9-S11.

## Discussion

Despite the clinical benefits of methadone for treating OUD and movements toward behavioral health parity in commercial and Medicaid health plans (eg, the Mental Health Parity and Addiction Equity Act, provisions in the Affordable Care Act), Medicare FFS surprisingly did not cover any OUD treatment services in OTPs until 2020. Our study showed that this nationwide expansion of Medicare coverage was followed by significantly increased Medicare insurance acceptance in OTPs, especially among for-profit OTPs. Additionally, although we found increases in the availability of OUD treatment services over the study period, including ongoing buprenorphine and ancillary services, these were not associated with the introduction of Medicare coverage. County-level access to OTPs for Medicare beneficiaries experienced significant policy-associated improvements with more counties having at least one Medicare-accepting-OTP and increases in the number of Medicare-accepting-OTPs, particularly in rural counties and those with a higher percentage of non-White residents. To our knowledge, this is the first study to focus on changes in ancillary OUD treatment offerings beyond methadone access. We build on prior studies by employing panel data, allowing for more rigorous analyses than previous cross-sectional studies. Furthermore, this study also sheds light on the role of OTP ownership and county racial composition, poverty rates, and rurality in the improvements in methadone access for Medicare beneficiaries.

Notably, although the share of for-profit OTPs accepting Medicare and offering certain OUD treatment services was quite low in 2018, by 2021, the Medicare acceptance rate among for-profits was comparable to nonprofit OTPs. However, the Medicare coverage change was not associated with a significant improvement in the availability of OUD treatment services studied. This finding is not consistent with our initial hypothesis. We had anticipated that this policy would encourage a wider range of OUD treatment offerings, given their inclusion in the bundled payments, and the higher profitability and potential for enhanced oversight associated with Medicare coverage. However, access to ancillary OUD treatment, such as comprehensive mental health assessment, remained limited, especially at for-profit OTPs. Previous literature has noted this lower availability in for-profit settings.^32-34^ This might be partially attributed to a lack of quality metrics since OTPs receive a fixed payment for treating Medicare patients, regardless of the extent or variety of ancillary services provided. Another potential explanation is selection issues. Specifically, while Medicare Advantage plans are required to reimburse services at OTPs, they mandate that their enrollees receive treatment at in-network OTPs. Consequently, these plans might increase profits by excluding OTPs offering more comprehensive OUD care if these OTPs attract the highest cost patients to their plan. Finally, if MOUD is generally underprovided and there is a high unmet need, it may be that regulators focus compliance efforts elsewhere.

Higher Medicare reimbursement rates may encourage for-profit OTPs to accept more dual eligibles, who are exceptionally vulnerable and are among the primary beneficiaries of the new Medicare OTP coverage.^21,35,36^ Given the limited capacity of nonprofit facilities, for-profit facilities would become increasingly important for OUD treatment. In 2020, 62% (191 625) of patients receiving methadone were treated in for-profit OTPs.^27^ With the SUD treatment system increasingly dominated by these facilities, aligning incentives to encourage access and the provision of high-quality care may be even more important than in the past. As alternative payment models gain traction in behavioral health, attention to these incentives will be critical.^37,38^ Ultimately, contracts under these payment models should incentivize high-quality care, requiring that facilities help patients remain engaged in treatment and improve outcomes.

The wider availability of ancillary services that we found is consistent with best practice guidance indicating that all patients should have access to these services. Importantly, however, ancillary services can become a barrier to lifesaving MOUD if mandated as a requirement for treatment.^39^ Per ASAM practice guidelines, the absence of ancillary services should not prevent or delay MOUD treatment.^17^ Thus, for OTPs to comply with best practice guidance, it is unequivocal that services are not a MOUD requirement and that patients are instead offered the option to utilize services in a shared decision-making process tailored to the patients’ individual needs and preferences. To improve access to and the quality of OUD care, the provision of ancillary services should be aligned with evidence and made more flexible.

Regarding county-level access, rural counties and those with a higher percentage of Black residents have historically had more limited access to SUD treatment facilities.^40^ We found that these counties experienced greater improved access after the Medicare coverage expansion among counties that saw changes in access to Medicare-accepting-OTPs. Specifically, rural counties were more likely to transition from having no Medicare-accepting-OTPs to having at least one and counties with a higher proportion of non-White residents saw a higher increase in the number of Medicare-accepting-OTPs. In a fentanyl era, improving access to MOUD among communities of color is especially important, given that in 2020, opioid overdose mortality among Black Americans exceeded that among White Americans for the first time since 1999.^10^ Specifically for the older population, the growth of opioid overdose mortality among older Black men was more than twice that of their White counterparts.^11^ Our finding that communities of color experienced higher increases in the number of Medicare-accepting-OTPs after 2020 suggests that coverage expansions may be one mechanism to mitigate the racial gap in OUD burden and overall OUD treatment access. Although the proportion of clients at OTPs receiving buprenorphine for OUD treatment, among those receiving MOUD, remains low, it has increased from 9.2% in 2020 to 12.1% in 2022.^41^ Thus, OTPs have the potential to expand access to buprenorphine for treating OUD.

Although OTPs are the only outpatient settings where all three non-interchangeable MOUD can be provided and play a critical role in OUD treatment access, reform is needed to lift the restrictive, punitive, and stigmatizing aspects of OTP regulations and practices. MOUD treatment pathways have historically been racialized with Black patients more likely to receive their MOUD from OTPs and White patients, especially those further from city centers, having reduced access to OTPs and more likely to receive their MOUD through office-based buprenorphine.^13^ In terms of improving overall OUD treatment access, it is encouraging to find increased OTP access across all counties with greater increases for communities of color. However, these changes may reinforce existing racial patterns with Black patients disproportionately subjected to treatment contingent on burdensome regulations. Multifaceted policy reforms are needed to mitigate access barriers and ensure that all MOUD and ancillary services are widely accessible for all communities. This should importantly include concerted policy advances toward lifting some elements of methadone treatment.^13^ One recent example of policy moving in this direction is the increased flexibility on take-home medications.^42^

Our study has several limitations. First, data on facilities’ Medicare acceptance and service availability were self-reported, potentially leading to reporting bias. We did not have data on the treatments that patients actually received and quality of care may vary. The difference in sample size between the full unbalanced and balanced panels could suggest measurement errors. Nevertheless, this was mitigated by including facility-fixed effects to evaluate within OTP changes and by confirming the robustness of our results with an alternative sample of OTPs excluded from the balanced panel. Fixed effects models only included counties that experienced changes in the number of Medicare-accepting-OTPs when describing differential changes in county-level access. Lastly, our findings on the Medicare coverage expansion may not be generalized to other health insurance programs.

## Conclusion

Opioid treatment programs are the only outpatient providers to dispense methadone for treating OUD and therefore play an essential role in OUD treatment. In 2020, Medicare FFS established weekly bundled payments for OUD treatment services delivered at OTPs, thereby covering methadone as an OUD treatment option for the first time. Our study showed that while this Medicare coverage expansion was associated with better OTP access for Medicare beneficiaries, racial patterns related to access to methadone treatment and limited availability of OUD treatment services at OTPs persist. By 2021, although 33% (from 32% in 2018 to 65% in 2021) of Medicare-only beneficiaries resided in counties that newly had at least one Medicare-accepting-OTP, 35% still lived in counties without such OTPs. To curb the ongoing opioid epidemic, continued Medicare reform is imperative to expand access to OUD treatment at OTPs.

## Supplementary Material

qxae076_Supplementary_Data
