# Supplementary material for: Access to treatment before and after Medicare coverage of opioid treatment programs
Source: Health Aff Sch. 2024 Jun 6;2(6):qxae076. doi: 10.1093/haschl/qxae076 (PMC11210307; doi:10.1093/haschl/qxae076)
Supplement: qxae076_Supplementary_Data [file qxae076_supplementary_data.zip › Figure Data.docx]

Data for Figure 1. Percentage of OTPs Accepting Medicare by Ownership (2018-2021)

| **Year** | **Full Analytic Sample (N=868)** | **For-profit OTPs (N=557)** | **Nonprofit OTPs (N=311)** |
| --- | --- | --- | --- |
| 2018 | 21.31 | 11.67 | 38.59 |
| 2019 | 22.24 | 12.39 | 39.87 |
| 2020 | 59.91 | 56.01 | 66.88 |
| 2021 | 80.76 | 78.46 | 84.89 |

Abbreviation: OTP, opioid treatment program.

Source: Authors’ analysis of National Directory of Drug and Alcohol Abuse Treatment Facilities data (2019-2022).

Data for Figure 2. Percentage of OTPs Offering OUD Treatment Services by Ownership (2018-2021)

| **OUD Service** | **Year** | **Full Analytic Sample (N=868)** | **For-profit OTPs (N=557)** | **Nonprofit OTPs (N=311)** |
| --- | --- | --- | --- | --- |
| Ongoing  Buprenorphine | 2018 | 69.35 | 71.10 | 66.24 |
|  | 2019 | 72.24 | 73.61 | 69.77 |
|  | 2020 | 75.35 | 75.94 | 74.28 |
|  | 2021 | 76.73 | 77.56 | 75.24 |
| HIV/AIDS Education and Support | 2018 | 81.68 | 77.74 | 88.75 |
|  | 2019 | 84.56 | 79.89 | 92.93 |
|  | 2020 | 86.52 | 82.05 | 94.53 |
|  | 2021 | 88.13 | 83.66 | 96.14 |
| Employment Services | 2018 | 46.20 | 44.17 | 49.84 |
|  | 2019 | 49.19 | 47.76 | 51.77 |
|  | 2020 | 50.58 | 48.65 | 54.02 |
|  | 2021 | 51.50 | 49.37 | 55.31 |
| Comprehensive Mental Health Assessment | 2018 | 26.61 | 11.13 | 54.34 |
|  | 2019 | 29.38 | 11.49 | 61.41 |
|  | 2020 | 30.99 | 13.82 | 61.74 |
|  | 2021 | 31.68 | 14.72 | 62.06 |

Abbreviations: OTP, opioid treatment program; OUD, opioid use disorder.

Source: Authors’ analysis of National Directory of Drug and Alcohol Abuse Treatment Facilities data (2019-2022).

Data for Figure 3. Adjusted Differential Changes in County-level Access to OTPs for Medicare Beneficiaries (2018-2021)

| **Group** | **β** | **CI Lower Bound** | **CI Upper Bound** |
| --- | --- | --- | --- |
| **A: Number of OTPs accepting Medicare** | | | |
| Top 25% Nonwhite | 2.1573 | 1.3445 | 2.9701 |
| > 10% below FPL | 1.4202 | 1.1166 | 1.7239 |
| Rural | 0.6014 | 0.2873 | 0.9156 |
| Reference Counties | 1.2987 | 1.0315 | 1.5660 |
| **B: Presence of OTPs accepting Medicare** | | | |
| Top 25% Nonwhite | 52.2836 | 39.9849 | 64.5823 |
| > 10% below FPL | 52.1188 | 41.5594 | 62.6782 |
| Rural | 77.1120 | 65.9093 | 88.3147 |
| Reference Counties | 59.8043 | 50.7864 | 68.8221 |

Abbreviations: OTP, opioid treatment program; CI, confidence interval; FPL, federal poverty level.

Source: Authors’ analysis of National Directory of Drug and Alcohol Abuse Treatment Facilities data (2019-2022).
