# Supplementary material for: Access to treatment before and after Medicare coverage of opioid treatment programs
Source: Health Aff Sch. 2024 Jun 6;2(6):qxae076. doi: 10.1093/haschl/qxae076 (PMC11210307; doi:10.1093/haschl/qxae076)
Supplement: qxae076_Supplementary_Data [file qxae076_supplementary_data.zip › Medicare OTP Supplement_May16.docx]

**Appendix to Access to Treatment Before and After Medicare Coverage of Opioid Treatment Programs**

1. **Methods**
   1. Statistical Analyses
2. **Tables**
   1. Table S1. No. (%) of OTPs Accepting Medicare by Ownership (2018-2021) for Figure 1
   2. Table S2. Regression Analyses Results for Figure 1: Coefficients for Changes in Medicare Acceptance (2018-2021) and Wald Tests Results
   3. Table S3. No. (%) of OTPs Offering OUD Treatment Services by Ownership (2018-2021) for Figure 2
   4. Table S4. Regression Analyses Results for Figure 2: Coefficients for Changes in Service Availability at OTPs (2018-2021) and Wald Tests Results
   5. Table S5. Access to OTPs in all US Counties in 2018 and 2021
   6. Table S6. Regression Analyses Results for Figure 3: Coefficients for Changes in County-level Access (2018-2021) and Wald Tests Results
   7. Table S7. Baseline Characteristics of OTPs in the Full Unbalanced Panel (2018)
   8. Table S8. Medicare Acceptance and Availability of OUD Services at OTPs in the Full Unbalanced Panel (2018-2021)
   9. Table S9. Robustness Check for Changes in Medicare Acceptance and Service Availability (2018-2021) Using an Alternative Sample
   10. Table S10. Robustness Check for Differential Changes in County-level Access (2018-2021) Using Alternative Models
   11. Table S11. Sensitivity Analyses for Differential Changes in Service Availability at OTPs (2018-2021) Using an Alternative Model
3. **Methods.** Statistical Analyses

**Estimating changes** **in Medicare acceptance and service availability at opioid treatment programs**

We employed linear regression models with facility-fixed effects to describe changes at opioid treatment programs (OTPs), including Medicare acceptance and the availability of four opioid use disorder (OUD) treatment services. We first assessed aggregate changes and then determined heterogeneity based on OTP ownership status by incorporating an interaction term as shown in the following equation:

$$\mathrm{Outcome}_{i,t}=\alpha\mathrm{Year}_{t}+\beta\mathrm{FP}_{i}\times\mathrm{Year}_{t}+\mathrm{OTP}_{i}+\varepsilon_{i,t} (1)$$

$\mathrm{Outcomes}_{i,t}$ are five binary variables: one indicating whether the OTP i accepted Medicare and four representing whether the OTP offered each of the four OUD treatments -- ongoing buprenorphine, HIV/AIDS education and support, employment services, and comprehensive mental health assessment services -- in year t. $\mathrm{Year}_{t}$ are indicators representing the years from 2018 to 2021. $\mathrm{FP}_{i}$ represents facility ownership and equals one if OTP i was for-profit. $\mathrm{OTP}_{i}$ are facility-fixed effects, controlling for time-invariant OTP characteristics. $\alpha$ and $\beta$ are the coefficients of interest. $\alpha$ indicates changes in the likelihood of nonprofit OTPs accepting Medicare or offering a certain type of the four OUD treatment services relative to 2018. $\beta$captures the differential changes in for-profit OTPs compared to nonprofit OTPs. These models used 3 472 OTP-year observations from the 868 OTPs in the balanced panel, while excluding the 104 OTP-year observations from 26 OTPs that switched their ownership during the study period.

**Estimating changes in county-level access to OTPs for Medicare beneficiaries**

We used linear regression models with county-fixed effects to describe aggregate changes in county-level access to OTPs for Medicare beneficiaries. We then determined the heterogeneity of the changes varying by county sociodemographic characteristics using the following equation:

$$\mathrm{Acc}_{c,t} = \mathrm{Sociodem}_{c}+ \alpha\mathrm{Year}_{t}+ \beta{\mathrm{Sociodem}_{c}\times Year}_{t}+\mathrm{State}_{s}+ \varepsilon_{c,t} (2)$$

$\mathrm{Acc}_{t,c}$ are access measures including a continuous variable denoting the number of OTPs accepting Medicare (Medicare-accepting-OTPs) and a dummy variable indicating the presence of Medicare-accepting-OTPs within county c in year t. $\mathrm{Sociodemo}_{c}$ is a vector representing county sociodemographic characteristics. It includes three dummy variables indicating whether county c had a nonwhite population in the top quartile (more than 27.02%) in 2018, whether the county had more than 10% residents living below the federal poverty line in 2018, and whether it was a rural county. $\mathrm{State}_{s}$ are state-fixed effects, accounting for time-invariant state characteristics. $\beta$ is the coefficient of interest, reflecting the heterogeneity of changes in county-level access to Medicare-accepting-OTPs associated with counties’ racial composition, poverty rates, and rurality. These models used a balanced panel of 3 112 county-year observations from 778 counties that experienced changes in access to Medicare-accepting-OTPs during the study period.

**Sensitivity analyses for changes in Medicare acceptance and service availability at OTPs**

We used the balanced panel and categorized OTPs into four types: OTPs that newly accepted Medicare within the study period from 2018 to 2021 (type 0, n=525); OTPs that never accepted Medicare (type 1, n=157); OTPs that always accepted Medicare (type 2, n=163); and the other OTPs that switched to not accepting Medicare or changed their Medicare acceptance status more than once (type 3, n=23). The following model was employed to determine the heterogeneity of changes in service availability associated with Medicare acceptance status:

$$\mathrm{Serv}_{i,t}=\mathrm{Type}_{i}+\alpha\mathrm{Year}_{t}+\beta\mathrm{Type}_{i}\times\mathrm{Year}_{t}+\mathrm{OTP}_{i}+\varepsilon_{i,t} (3)$$

$\mathrm{Type}_{i}$ represents the type of OTP i with a reference group of the type 0 OTPs. $\beta$ is the coefficient of interest, capturing the heterogeneity of changes across different types of OTPs. 92 OTP-year observations from the 23 type 3 OTPs were excluded from our analyses.

1. **Tables**

Table S1. No. (%) of OTPs Accepting Medicare by Ownership (2018-2021) for Figure 1

| **Year** | **Full Analytic Sample (N=868)** | **For-profit OTPs (N=557)** | **Nonprofit OTPs (N=311)** | **OTPs Excluded due to Ownership Switch (N=26)** |
| --- | --- | --- | --- | --- |
| 2018 | 185 (21.31) | 65 (11.67) | 120 (38.59) | 6 (23.08) |
| 2019 | 193 (22.24) | 69 (12.39) | 124 (39.87) | 5 (19.23) |
| 2020 | 520 (59.91) | 312 (56.01) | 208 (66.88) | 14 (53.85) |
| 2021 | 701 (80.76) | 437 (78.46) | 264 (84.89) | 18 (69.23) |

Abbreviation: OTP, opioid treatment program.

Source: Authors’ analysis of National Directory of Drug and Alcohol Abuse Treatment Facilities data (2019-2022).

Table S2. Regression Analyses Results for Figure 1: Coefficients for Changes in Medicare Acceptance (2018-2021) and Wald Tests Results

a. Coefficients for Changes in Medicare Acceptance among For-profit and Nonprofit OTPs (2018-2021)

| **Variable** | **Medicare Acceptance**  **(β [95% CI])** |
| --- | --- |
| **A: Aggregate** |  |
| Year2019 | 0.009 (-0.006-0.02) |
| Year2020 | 0.39 (0.35-0.42)^a^ |
| Year2021 | 0.59 (0.56-0.63)^a^ |
| Facility F.E. | Yes |
| Observations | 3 472 |
| R^2^ | 0.69 |
| **B: Ownership Heterogeneity** |  |
| Year2019 | 0.01 (-0.02-0.05) |
| Year2020 | 0.28 (0.23-0.34)^a^ |
| Year2021 | 0.46 (0.40-0.52)^a^ |
| FP × Year2019 | -0.006 (-0.04-0.03) |
| FP × Year2020 | 0.16 (0.09-0.23)^a^ |
| FP × Year2021 | 0.21 (0.13-0.28)^a^ |
| Facility F.E. | Yes |
| Intercept | Yes |
| Observations | 3 472 |
| R^2^ | 0.67 |

Abbreviations: OTP, opioid treatment program; CI, confidence interval; F.E., fixed effects; FP, whether an OTP was for-profit.

Source: Authors’ analysis of National Directory of Drug and Alcohol Abuse Treatment Facilities data (2019-2022).

^a^ P < .001.

b. Wald Tests for the Coefficients for Changes in Medicare Acceptance

| **Constraint** | **Chi-squared (P-value)** |
| --- | --- |
| **A: Aggregate** |  |
| Year2019 - Year2018 = Year2020 - Year2019 | 376.46 (< 0.001) |
| Year2020 - Year2019 = Year2021 - Year2020 | 42.59 (< 0.001) |
| Year2019 - Year2018 = Year2021 - Year2020 | 140.55 (< 0.001) |
| **B: Ownership Heterogeneity** |  |
| Year2019 - Year2018 = Year2020 - Year2019 | 64.19 (< 0.001) |
| Year2020 - Year2019 = Year2021 - Year2020 | 5.30 (0.021) |
| Year2019 - Year2018 = Year2021 - Year2020 | 30.19 (< 0.001) |
| (FP × Year2019 + Year2019) - (FP × Year2018 + Year2018) = (FP × Year2020 + Year2020) - (FP × Year2019 + Year2019) | 346.35 (< 0.001) |
| (FP × Year2020 + Year2020) - (FP × Year2019 + Year2019) = (FP × Year2021 + Year2021) - (FP × Year2020 + Year2020) | 39.78 (< 0.001) |
| (FP × Year2019 + Year2019) - (FP × Year2018 + Year2018) = (FP × Year2021 + Year2021) - (FP × Year2020 + Year2020) | 118.97 (< 0.001) |

Abbreviation: FP, whether an OTP was for-profit.

Source: Authors’ analysis of National Directory of Drug and Alcohol Abuse Treatment Facilities data (2019-2022).

Table S3. No. (%) of OTPs Offering OUD Treatment Services by Ownership (2018-2021) for Figure 2

| **OUD Service** | **Year** | **Full Analytic Sample (N=868)** | **For-profit OTPs (N=557)** | **Nonprofit OTPs (N=311)** | **OTPs Excluded due to Ownership Switch (N=26)** |
| --- | --- | --- | --- | --- | --- |
| Ongoing  Buprenorphine | 2018 | 602 (69.35) | 396 (71.10) | 206 (66.24) | 20 (76.92) |
|  | 2019 | 627 (72.24) | 410 (73.61) | 217 (69.77) | 21 (80.77) |
|  | 2020 | 654 (75.35) | 423 (75.94) | 231 (74.28) | 18 (69.23) |
|  | 2021 | 666 (76.73) | 432 (77.56) | 234 (75.24) | 17 (65.38) |
| HIV/AIDS Education and Support | 2018 | 709 (81.68) | 433 (77.74) | 276 (88.75) | 15 (57.69) |
|  | 2019 | 734 (84.56) | 445 (79.89) | 289 (92.93) | 17 (65.38) |
|  | 2020 | 751 (86.52) | 457 (82.05) | 294 (94.53) | 20 (76.92) |
|  | 2021 | 765 (88.13) | 466 (83.66) | 299 (96.14) | 18 (69.23) |
| Employment Services | 2018 | 401 (46.20) | 246 (44.17) | 155 (49.84) | 8 (30.77) |
|  | 2019 | 427 (49.19) | 266 (47.76) | 161 (51.77) | 12 (46.15) |
|  | 2020 | 439 (50.58) | 271 (48.65) | 168 (54.02) | 14 (53.85) |
|  | 2021 | 447 (51.50) | 275 (49.37) | 172 (55.31) | 13 (50.00) |
| Comprehensive Mental Health Assessment | 2018 | 231 (26.61) | 62 (11.13) | 169 (54.34) | 1 (3.85) |
|  | 2019 | 255 (29.38) | 64 (11.49) | 191 (61.41) | 3 (11.54) |
|  | 2020 | 269 (30.99) | 77 (13.82) | 192 (61.74) | 3 (11.54) |
|  | 2021 | 275 (31.68) | 82 (14.72) | 193 (62.06) | 5 (19.23) |

Abbreviations: OTP, opioid treatment program; OUD, opioid use disorder.

Source: Authors’ analysis of National Directory of Drug and Alcohol Abuse Treatment Facilities data (2019-2022).

Table S4. Regression Analyses Results for Figure 2: Coefficients for Changes in Service Availability at OTPs (2018-2021) and Wald Tests Results

a. Coefficients for Changes in Service Availability among For-profit and Nonprofit OTPs (2018-2021)

| **Variable** | **Ongoing**  **buprenorphine**  **(β [95% CI])** | **HIV or AIDS education and support**  **(β [95% CI])** | **Employment**  **services**  **(β [95% CI])** | **Comprehensive mental health assessment**  **(β [95% CI])** |
| --- | --- | --- | --- | --- |
| **A: Aggregate** |  |  |  |  |
| Year2019 | 0.03 (0.01 - 0.05)^a^ | 0.03 (0.01 - 0.04)^b^ | 0.03 (0.009 - 0.05)^a^ | 0.03 (0.01 - 0.04)^b^ |
| Year2020 | 0.06 (0.04 - 0.08)^b^ | 0.05 (0.03 - 0.07)^b^ | 0.04 (0.02 - 0.07)^b^ | 0.04 (0.03 - 0.06)^b^ |
| Year2021 | 0.07 (0.05 - 0.10)^b^ | 0.07 (0.04 - 0.09)^b^ | 0.05 (0.02 - 0.08)^b^ | 0.05 (0.03 - 0.07)^b^ |
| Facility F.E. | Yes | Yes | Yes | Yes |
| Observations | 3 472 | 3 472 | 3 472 | 3 472 |
| R^2^ | 0.82 | 0.79 | 0.83 | 0.88 |
| **B: Ownership Heterogeneity** |  |  |  |  |
| Year2019 | 0.04 (0.0004 - 0.07)^c^ | 0.04 (0.02 - 0.07)^a^ | 0.02 (-0.02 - 0.06) | 0.07 (0.04 - 0.10)^b^ |
| Year2020 | 0.08 (0.04 - 0.12)^b^ | 0.06 (0.03 - 0.09)^b^ | 0.04 (-0.0003 - 0.08) | 0.07 (0.04 - 0.11)^b^ |
| Year2021 | 0.09 (0.05 - 0.13)^b^ | 0.07 (0.04 - 0.11)^b^ | 0.06 (0.006 - 0.10)^c^ | 0.08 (0.03 - 0.12)^b^ |
| FP × Year2019 | -0.01 (-0.05 - 0.03) | -0.02 (-0.05 - 0.01) | 0.02 (-0.03 - 0.06) | -0.07 (-0.10 - -0.03)^b^ |
| FP × Year2020 | -0.03 (-0.08 - 0.02) | -0.01 (-0.06 - 0.03) | 0.003 (-0.05 - 0.06) | -0.05 (-0.09 - -0.006)^c^ |
| FP × Year2021 | -0.03 (-0.08 - 0.03) | -0.01 (-0.06 - 0.03) | -0.003 (-0.06 - 0.06) | -0.04. (-0.09 - 0.008) |
| Facility F.E. | Yes | Yes | Yes | Yes |
| Intercept | Yes | Yes | Yes | Yes |
| Observations | 3 472 | 3 472 | 3 472 | 3 472 |
| R^2^ | 0.82 | 0.79 | 0.83 | 0.89 |

Abbreviations: OTP, opioid treatment program; CI, confidence interval; F.E., fixed effects; FP, whether an OTP was for-profit.

Source: Authors’ analysis of National Directory of Drug and Alcohol Abuse Treatment Facilities data (2019-2022).

^a^ P < .01.

^b^ P < .001.

^c^ P < .05.

b. Wald Tests for the Coefficients for Changes in Service Availability at OTPs

| **Constraint** | **Chi-squared (P-value)** | **Chi-squared (P-value)** | **Chi-squared (P-value)** | **Chi-squared (P-value)** |
| --- | --- | --- | --- | --- |
| **A: Aggregate** |  |  |  |  |
| Year2019 - Year2018 = Year2020 - Year2019 | 0.02 (0.878) | 0.74 (0.389) | 1.26 (0.263) | 1.25 (0.264) |
| Year2020 - Year2019 = Year2021 - Year2020 | 1.64 (0.200) | 0.10 (0.751) | 0.15 (0.701) | 0.76 (0.383) |
| Year2019 - Year2018 = Year2021 - Year2020 | 1.40 (0.237) | 1.33 (0.249) | 2.38 (0.123) | 3.87 (0.049) |
| **B: Ownership Heterogeneity** |  |  |  |  |
| Year2019 - Year2018 = Year2020 - Year2019 | 0.11 (0.742) | 2.01 (0.157) | 0.02 (0.901) | 8.87 (0.003) |
| Year2020 - Year2019 = Year2021 - Year2020 | 1.71 (0.191) | 0.00 (1.000) | 0.21 (0.648) | 0.00 (1.000) |
| Year2019 - Year2018 = Year2021 - Year2020 | 1.19 (0.276) | 2.01 (0.157) | 0.07 (0.790) | 8.52 (0.004) |
| (FP × Year2019 + Year2019) - (FP × Year2018 + Year2018) = (FP × Year2020 + Year2020) - (FP × Year2019 + Year2019) | 0.01 (0.915) | 0.00 (1.000) | 2.48 (0.116) | 4.19 (0.041) |
| (FP × Year2020 + Year2020) - (FP × Year2019 + Year2019) = (FP × Year2021 + Year2021) - (FP × Year2020 + Year2020) | 0.24 (0.623) | 0.16 (0.686) | 0.02 (0.901) | 1.45 (0.228) |
| (FP × Year2019 + Year2019) - (FP × Year2018 + Year2018) = (FP × Year2021 + Year2021) - (FP × Year2020 + Year2020) | 0.37 (0.542) | 0.15 (0.697) | 3.21 (0.073) | 0.29 (0.591) |
| Outcome | Ongoing  buprenorphine | HIV or AIDS education and support | Employment services | Comprehensive mental health assessment |

Abbreviations: OTP, opioid treatment program; FP, whether an OTP was for-profit.

Source: Authors’ analysis of National Directory of Drug and Alcohol Abuse Treatment Facilities data (2019-2022).

Table S5. Access to OTPs in all US Counties in 2018 and 2021

| **Category** | **2018** | | **2021** | |
| --- | --- | --- | --- | --- |
|  | **No.** | **Percentage^a^** | **No.** | **Percentage^a^** |
| Counties with OTPs^b^ | 599 | 18.19% | 689 | 21.00% |
| Medicare beneficiaries^c^ | 40 354 273 | 67.82% | 44 321 598 | 69.95% |
| Medicare-only beneficiaries^d^ | 32 355 887 | 67.67% | 35 318 895 | 69.81% |
| Counties without OTPs | 2 694 | 81.81% | 2 592 | 79.00% |
| Medicare beneficiaries | 19 148 513 | 32.18% | 19 042 426 | 30.05% |
| Medicare-only beneficiaries | 15 458 538 | 32.33% | 15 274 642 | 30.19% |
| Counties with OTPs accepting Medicare | 165 | 5.01% | 588 | 17.92% |
| Medicare beneficiaries | 19 384 782 | 32.58% | 41 359 742 | 65.27% |
| Medicare-only beneficiaries | 15 178 874 | 31.75% | 32 957 854 | 65.14% |
| Counties without OTPs accepting Medicare | 3 128 | 94.99% | 2 693 | 82.08% |
| Medicare beneficiaries | 40 118 004 | 67.42% | 22 004 282 | 34.73% |
| Medicare-only beneficiaries | 32 635 551 | 68.25% | 17 635 683 | 34.86% |

Abbreviations: OTP, opioid treatment program.

Source: Authors’ analysis of the 2019 and 2022 National Directory of Drug and Alcohol Abuse Treatment Facilities data.

^a^ The denominators were the total number of US counties (3 293 in 2018 and 3 281 in 2021), the total number of Medicare beneficiaries (59 502 786 in 2018 and 63 364 024 in 2021), or the total number of Medicare-only beneficiaries (47 814 425 in 2018 and 50 593 537 in 2021).

^b^ OTPs in the full unbalanced longitudinal sample.

^c^ Beneficiaries who lived in those counties and enrolled in Medicare, including dual-eligibles.

^d^ Beneficiaries who lived in those counties and received health insurance coverage exclusively from Medicare.

Table S6. Regression Analyses Results for Figure 3: Coefficients for Changes in County-level Access (2018-2021) and Wald Tests Results

a. Coefficients for Changes in County-level Access to OTPs for Medicare Beneficiaries (2018-2021)

| **Variable** | **Number of OTPs accepting Medicare**  **(β [95% CI])** | **Presence of OTPs accepting Medicare**  **(β [95% CI])** |
| --- | --- | --- |
| **A: Aggregate** |  |  |
| Year2019 | 0.06 (0.02 – 0.09)^c^ | 0.02 (0.003 – 0.04)^d^ |
| Year2020 | 0.80 (0.70 – 0.90)^c^ | 0.40 (0.36 – 0.44)^c^ |
| Year2021 | 1.30 (1.10 – 1.46)^c^ | 0.56 (0.52 – 0.59)^c^ |
| County F.E. | Yes | Yes |
| Observations | 3 112 | 3 112 |
| R^2^ | 0.80 | 0.64 |
| **B:** **Sociodemographic Heterogeneity** |  |  |
| Year2019 | 0.06 (0.010 – 0.10)^d^ | 0.05 (-0.006 – 0.11) |
| Year2020 | 0.41 (0.33 – 0.50)^c^ | 0.75 (0.56 – 0.94)^c^ |
| Year2021 | 0.60 (0.51 – 0.69)^c^ | 1.30 (1.03 – 1.57)^c^ |
| Top nonwhite population quartile^a^ | 0.17 (0.08 – 0.26)^c^ | 1.01 (0.34 – 1.67)^e^ |
| Rural | -0.15 (-0.25 - -0.06)^e^ | -0.36 (-0.63 - -0.10)^e^ |
| > 10% below FPL^b^ | 0.07 (-0.02 – 0.16) | 0.27 (-0.09 – 0.62) |
| Year2019 × Top nonwhite population quartile | -0.07 (-0.12 - -0.02)^e^ | -0.07 (-0.17 – 0.03) |
| Year2020 × Top nonwhite population quartile | -0.003 (-0.09 – 0.09) | 0.40 (-0.02 – 0.83) |
| Year2021 × Top nonwhite population quartile | -0.07 (-0.20 – 0.05) | 0.86 (0.05 – 1.67)^d^ |
| Year2019 × Rural | 0.01 (-0.06 – 0.09) | -0.02 (-0.09 – 0.04) |
| Year2020 × Rural | 0.11 (-0.007 – 0.23) | -0.44 (-0.66 - -0.21)^c^ |
| Year2021 × Rural | 0.17 (0.06 – 0.29)^e^ | -0.70 (-1.01 - -0.38)^c^ |
| Year2019 × > 10% below FPL | -0.007 (-0.05 – 0.04) | 0.06 (-0.02 – 0.14) |
| Year2020 × > 10% below FPL | 0.02 (-0.07 – 0.10) | 0.24 (0.02 – 0.46)^d^ |
| Year2021 × > 10% below FPL | -0.08 (-0.18 – 0.03) | 0.12 (-0.18 – 0.43) |
| State F.E. | Yes | Yes |
| Observations | 2 400 | 2 400 |
| R^2^ | 0.37 | 0.23 |

Abbreviations: OTP, opioid treatment program; CI, confidence interval; F.E., fixed effects; FPL, federal poverty level.

Source: Authors’ analysis of National Directory of Drug and Alcohol Abuse Treatment Facilities data (2019-2022).

Notes: This analytic sample included 3 112 county-year observations from 778 counties that experienced changes in access to Medicare-accepting-OTPs. In the heterogeneity analyses, 712 observations from 178 counties were omitted due to missing information on county socioeconomic characteristics.

^a^ Counties with more than 27.02% nonwhite residents in 2018.

^b^ Counties with more than 10% residents living below the federal poverty line in 2018.

^c^ P < .001.

^d^ P < .05.

^e^ P < .01.

b. Wald Tests for the Coefficients for Changes in County-level Access to OTPs

| **Constraint** | **Chi-squared (P-value)** | **Chi-squared (P-value)** |
| --- | --- | --- |
| Year2019 – Year2018 = Year2020 – Year2019 | 214.98 (< 0.001) | 251.79 (< 0.001) |
| Year2020 – Year2019 = Year2021 – Year2020 | 22.09 (< 0.001) | 51.78 (< 0.001) |
| Year2019 – Year2018 = Year2021 – Year2020 | 99.03 (< 0.001) | 37.72 (< 0.001) |
| Outcome | Number of OTPs accepting Medicare | Presence of OTPs accepting Medicare |

Abbreviation: OTP, opioid treatment program.

Source: Authors’ analysis of National Directory of Drug and Alcohol Abuse Treatment Facilities data (2019-2022).

Table S7. Baseline Characteristics of OTPs in the Full Unbalanced Panel (2018)

|  | **Unbalanced Sample, No. (%)** | | | | **OTPs Excluded from the Balanced Sample due to Not Listed in All Four Years, No. (%)** | | | |
| --- | --- | --- | --- | --- | --- | --- | --- | --- |
| **Characteristic** | **OTPs with Stable Ownership** | **Always For-profit OTPs** | **Always Nonprofit OTPs** | **OTPs with Switched Ownership** | **OTPs with Stable Ownership** | **Always For-profit OTPs** | **Always Nonprofit OTPs** | **OTPs with Switched Ownership** |
| Total OTPs, No. | 2 209 | 1 385 | 824 | 37 | 1 341 | 828 | 513 | 11 |
| For-profit | 1 385 (62.70) | 1 385 (100.00) | 0 (0.00) | NA | 828 (61.74) | 828 (100.00) | 0 (0.00) | NA |
| Accepting Medicare^a^ | 487 (22.05) | 173 (12.49) | 327 (39.74) | 9 (24.14) | 314 (23.45) | 116 (14.00) | 216 (42.11) | 4 (33.33) |
| Treatment services |  |  |  |  |  |  |  |  |
| Ongoing buprenorphine | 1 521 (68.86) | 974 (70.36) | 545 (66.09) | 27 (72.41) | 911 (67.92) | 571 (69.00) | 338 (65.79) | 4 (33.33) |
| HIV or AIDS education and support | 1 750 (79.24) | 1 041 (75.15) | 715 (86.83) | 22 (58.62) | 1 000 (74.56) | 582 (70.33) | 425 (82.89) | 7 (66.67) |
| Employment services | 999 (45.23) | 598 (43.17) | 404 (49.03) | 11 (31.03) | 581 (43.36) | 342 (41.33) | 243 (47.37) | 4 (33.33) |
| Comprehensive mental health assessment | 582 (26.36) | 160 (11.55) | 443 (53.78) | 1 (3.45) | 347 (25.88) | 102 (12.33) | 270 (52.63) | 0 (0.00) |
| County Characteristics |  |  |  |  |  |  |  |  |
| Rural | 323 (14.60) | 197 (14.24) | 125 (15.21) | 5 (13.51) | 231 (17.20) | 121 (14.65) | 109 (21.33) | 2 (18.18) |
| Top nonwhite population quartile^b^ | 868 (39.31) | 517 (37.36) | 351 (42.61) | 25 (67.57) | 504 (37.60) | 309 (37.26) | 196 (38.17) | 8 (72.73) |
| > 10% below FPL^c^ | 1 670 (75.62) | 1 040 (75.06) | 631 (76.60) | 30 (81.82) | 1 018 (75.90) | 625 (75.43) | 394 (76.73) | 9 (80.00) |
| Region |  |  |  |  |  |  |  |  |
| Northeast | 567 (25.69) | 237 (17.11) | 332 (40.30) | 6 (16.67) | 355 (26.44) | 146 (17.64) | 210 (40.84) | 1 (9.09) |
| Midwest | 415 (18.78) | 244 (17.62) | 171 (20.77) | 5 (13.89) | 277 (20.69) | 169 (20.44) | 108 (21.12) | 3 (27.27) |
| South | 784 (35.47) | 608 (43.87) | 174 (21.14) | 14 (38.89) | 463 (34.52) | 348 (41.97) | 114 (22.31) | 5 (45.45) |
| West | 443 (20.06) | 296 (21.39) | 147 (17.80) | 11 (30.56) | 246 (18.35) | 165 (19.95) | 81 (15.74) | 2 (18.18) |

Abbreviations: OTP, opioid treatment program; FPL, federal poverty level.

Source: Authors’ analysis of the 2019 National Directory of Drug and Alcohol Abuse Treatment Facilities data.

^a^ OTPs that reported accepting Medicare insurance. This may include traditional Medicare payments for services not related to opioid use disorder treatment, and Medicare Advantage payments for treatment services related to opioid use disorder.

^b^ Counties with more than 27.02% nonwhite residents in 2018.

^b^ Counties with more than 10% residents living below the federal poverty line in 2018.

Table S8. Medicare Acceptance and Availability of OUD Services at OTPs in the Full Unbalanced Panel (2018-2021)

| **Outcome** | **Year** | **Balanced Sample, No. (%) (N=868)** | **OTPs Excluded due to Ownership Switch, No. (%) (N=26)** | **OTPs Excluded due to Not Listed in All Four Years, No. (%) (N=1 352)** | **Total OTPs, No. (%) (N=2 246)** |
| --- | --- | --- | --- | --- | --- |
| Medicare Acceptance | 2018 | 187 (21.31) | 6 (23.08) | 318 (23.52) | 496 (22.09) |
|  | 2019 | 195 (22.24) | 5 (19.23) | 352 (26.00) | 530 (23.61) |
|  | 2020 | 526 (59.91) | 14 (53.85) | 866 (64.07) | 1 381 (61.48) |
|  | 2021 | 709 (80.76) | 18 (69.23) | 946 (69.94) | 1 692 (75.33) |
| Ongoing  Buprenorphine | 2018 | 609 (69.35) | 20 (76.92) | 915 (67.69) | 1 548 (68.94) |
|  | 2019 | 634 (72.24) | 21 (80.77) | 954 (70.55) | 1 612 (71.75) |
|  | 2020 | 662 (75.35) | 18 (69.23) | 1 041 (76.99) | 1 705 (75.90) |
|  | 2021 | 674 (76.73) | 17 (65.38) | 986 (72.90) | 1 678 (74.70) |
| HIV/AIDS Education and Support | 2018 | 717 (81.68) | 15 (57.69) | 1 007 (74.51) | 1 770 (78.80) |
|  | 2019 | 742 (84.56) | 17 (65.38) | 1 072 (79.27) | 1 846 (82.20) |
|  | 2020 | 760 (86.52) | 20 (76.92) | 1 090 (80.63) | 1 886 (83.98) |
|  | 2021 | 774 (88.13) | 18 (69.23) | 1 048 (77.51) | 1 857 (82.69) |
| Employment Services | 2018 | 406 (46.20) | 8 (30.77) | 585 (43.30) | 1 009 (44.92) |
|  | 2019 | 432 (49.19) | 12 (46.15) | 686 (50.73) | 1 117 (49.72) |
|  | 2020 | 444 (50.58) | 14 (53.85) | 694 (51.32) | 1 144 (50.93) |
|  | 2021 | 452 (51.50) | 13 (50.00) | 710 (52.54) | 1 167 (51.98) |
| Comprehensive Mental Health Assessment | 2018 | 234 (26.61) | 1 (3.85) | 348 (25.71) | 581 (25.87) |
|  | 2019 | 258 (29.38) | 3 (11.54) | 401 (29.64) | 655 (29.16) |
|  | 2020 | 272 (30.99) | 3 (11.54) | 390 (28.81) | 669 (29.77) |
|  | 2021 | 278 (31.68) | 5 (19.23) | 672 (49.70) | 904 (40.25) |

Abbreviations: OUD, opioid use disorder; OTP, opioid treatment program.

Source: Authors’ analysis of National Directory of Drug and Alcohol Abuse Treatment Facilities data (2019-2022).

Table S9. Robustness Check for Changes in Medicare Acceptance and Service Availability (2018-2021) Using an Alternative Sample

| **Variable** | **Medicare Acceptance**  **(β [95% CI])** | **Ongoing buprenorphine**  **(β [95% CI])** | **HIV or AIDS education and support**  **(β [95% CI])** | **Employment services**  **(β [95% CI])** | **Comprehensive mental health assessment**  **(β [95% CI])** |
| --- | --- | --- | --- | --- | --- |
| **A: Aggregate** |  |  |  |  |  |
| Year2019 | 0.04. (-0.001-0.07) | -0.0008 (-0.03-0.03) | 0.02 (-0.02-0.05) | 0.08 (0.04-0.12)^a^ | 0.01 (-0.01-0.04) |
| Year2020 | 0.38 (0.33-0.44)^a^ | 0.05 (0.02-0.09)^b^ | 0.04 (-0.006-0.08) | 0.09 (0.04-0.14)^a^ | 0.03 (-0.010-0.06) |
| Year2021 | 0.55 (0.49-0.62)^a^ | 0.06 (0.01-0.10)^c^ | 0.05 (-0.0010-0.11) | 0.14 (0.08-0.19)^a^ | 0.06 (0.02-0.11)^b^ |
| Facility F.E. | Yes | Yes | Yes | Yes | Yes |
| Observations | 2 425 | 2 425 | 2 425 | 2 425 | 2 425 |
| R^2^ | 0.81 | 0.89 | 0.87 | 0.90 | 0.92 |
| **B: Ownership Heterogeneity** | |  |  |  |  |
| Year2019 | 0.04 (-0.04-0.12) | 0.01 (-0.04-0.07) | -0.05 (-0.13-0.02) | 0.05 (-0.006-0.10) | 0.03 (-0.03-0.09) |
| Year2020 | 0.21 (0.11-0.31)^a^ | 0.07 (0.009-0.14)^c^ | -0.03 (-0.12-0.05) | 0.07 (0.01-0.13)^c^ | 0.04 (-0.03-0.11) |
| Year2021 | 0.39 (0.26-0.52)^a^ | 0.07 (-0.007-0.15) | -0.01 (-0.12-0.09) | 0.06 (-0.01-0.12) | 0.06 (-0.04-0.16) |
| FP × Year2019 | -0.009 (-0.10-0.08) | -0.02 (-0.09-0.05) | 0.10 (0.01-0.19)^c^ | 0.05 (-0.03-0.13) | -0.02 (-0.09-0.04) |
| FP × Year2020 | 0.26 (0.14-0.38)^a^ | -0.03 (-0.11-0.05) | 0.10 (0.008-0.20)^c^ | 0.03 (-0.05-0.11) | -0.02 (-0.10-0.06) |
| FP × Year2021 | 0.24 (0.10-0.39)^b^ | -0.02 (-0.12-0.07) | 0.10 (-0.02-0.22) | 0.11 (0.01-0.21)^c^ | 0.006 (-0.11-0.12) |
| Facility F.E. | Yes | Yes | Yes | Yes | Yes |
| Intercept | Yes | Yes | Yes | Yes | Yes |
| Observations | 2 425 | 2 425 | 2 425 | 2 425 | 2 425 |
| R^2^ | 0.81 | 0.89 | 0.87 | 0.90 | 0.93 |

Abbreviations: CI, confidence interval; F.E.: fixed effects. FP: whether an OTP was for-profit.

Source: Authors’ analysis of National Directory of Drug and Alcohol Abuse Treatment Facilities data (2019-2022).

Notes: This alternative sample included 2 425 observations from 1 341 OTPs. These OTPs had consistent ownership but were excluded from the balanced longitudinal sample because they were not listed in all four years' directories.

^a^ P < .001.

^b^ P < .01.

^c^ P < .05.

Table S10. Robustness Check for Differential Changes in County-level Access (2018-2021) Using Alternative Models

a. Robustness Check for Differential Changes in the Number of OTPs Accepting Medicare within Counties (2018-2021) Using Alternative Models

| **Variable** | **Model (1)**  **(β [95% CI])** | **Model (2)**  **(β [95% CI])** | **Model (3)**  **(β [95% CI])** |
| --- | --- | --- | --- |
| Year2019 | 0.09 (0.04 - 0.14)^c^ | 0.04 (-0.02 - 0.10) | 0.05 (-0.006 - 0.11) |
| Year2020 | 0.89 (0.73 - 1.04)^d^ | 0.84 (0.63 - 1.05)^d^ | 0.70 (0.52 - 0.88)^d^ |
| Year2021 | 1.40 (1.2 - 1.59)^d^ | 1.5 (1.2 - 1.79)^d^ | 1.2 (0.96 - 1.5)^d^ |
| Top nonwhite population quartile^a^ | 0.89 (0.28 - 1.50)^c^ | NA | 1.0 (0.36 - 1.7)^c^ |
| Rural | -0.19 (-0.38 - -0.002)^e^ | -0.48 (-0.77 - -0.18)^c^ | NA |
| > 10% below FPL^b^ | NA | -0.047 (-0.22 - 0.12) | 0.005 (-0.164 - 0.174) |
| Year2019 × Top nonwhite population quartile | -0.06 (-0.14 - 0.03) | NA | -0.07 (-0.16 - 0.03) |
| Year2020 × Top nonwhite population quartile | 0.37 (-0.003 - 0.75) | NA | 0.43 (-0.0001 - 0.87) |
| Year2021 × Top nonwhite population quartile | 0.70 (-0.02 - 1.42) | NA | 0.91 (0.08 - 1.73)^e^ |
| Year2019 × Rural | -0.05 (-0.10 - -0.001)^e^ | -0.01 (-0.08 - 0.05) | NA |
| Year2020 × Rural | -0.55 (-0.73 - -0.37)^d^ | -0.47 (-0.71 - -0.22)^d^ | NA |
| Year2021 × Rural | -0.73 (-1.0 - -0.45)^d^ | -0.77 (-1.1 - -0.41)^d^ | NA |
| Year2019 × > 10% below FPL | NA | 0.06 (-0.02 - 0.13) | 0.06 (-0.02 - 0.14) |
| Year2020 × > 10% below FPL | NA | 0.26 (0.05 - 0.48)^e^ | 0.20 (-0.02 - 0.42) |
| Year2021 × > 10% below FPL | NA | 0.18 (-0.10 - 0.46) | 0.06 (-0.24 - 0.36) |
| State F.E. | Yes | Yes | Yes |
| Observations | 3 072 | 2 416 | 2 400 |
| R^2^ | 0.22 | 0.19 | 0.22 |

Abbreviations: OTP, opioid treatment program; CI, confidence interval; FPL, federal poverty level; F.E.: fixed effects.

Source: Authors’ analysis of National Directory of Drug and Alcohol Abuse Treatment Facilities data (2019-2022).

Notes: This analytic sample included 3 112 county-year observations from 778 counties that experienced changes in access to Medicare-accepting-OTPs. In model 1, 40 observations from 10 counties were excluded due to missing information on racial composition. In Model 2, 696 observations from 174 counties were omitted because of missing information on county poverty rates. In Model 3, 712 observations from 178 counties were excluded due to missing data on both county poverty rate and racial composition.

^a^ Counties with more than 27.02% nonwhite residents in 2018.

^b^ Counties with more than 10% residents living below the federal poverty line in 2018.

^c^ P < .01.

^d^ P < .001.

^e^ P < .05.

b. Robustness Check for Differential Changes in the Presence of OTPs Accepting Medicare within Counties (2018-2021) Using Alternative Models

| **Variable** | **Model (1)**  **(β [95% CI])** | **Model (2)**  **(β [95% CI])** | **Model (3)**  **(β [95% CI])** |
| --- | --- | --- | --- |
| Year2019 | 0.05 (0.01 - 0.08)^c^ | 0.04 (-0.009 - 0.09) | 0.06 (0.01 - 0.10)^e^ |
| Year2020 | 0.42 (0.36 - 0.47)^d^ | 0.41 (0.34 - 0.49)^d^ | 0.43 (0.35 - 0.51)^d^ |
| Year2021 | 0.55 (0.49 - 0.61)^d^ | 0.58 (0.49 - 0.67)^d^ | 0.62 (0.53 - 0.70)^d^ |
| Top nonwhite population quartile^a^ | 0.15 (0.07 - 0.24)^d^ | NA | 0.18 (0.09 - 0.28)^d^ |
| Rural | -0.14 (-0.20 - -0.07)^d^ | -0.17 (-0.27 - -0.07)^d^ | NA |
| > 10% below FPL^b^ | NA | 0.09 (0.002 - 0.18)^e^ | 0.06 (-0.04 - 0.15) |
| Year2019 × Top nonwhite population quartile | -0.06 (-0.11 - -0.02)^c^ | NA | -0.07 (-0.12 - -0.02)^c^ |
| Year2020 × Top nonwhite population quartile | 0.004 (-0.08 - 0.08) | NA | -0.01 (-0.10 - 0.08) |
| Year2021 × Top nonwhite population quartile | -0.10 (-0.18 - -0.02)^e^ | NA | -0.09 (-0.21 - 0.04) |
| Year2019 × Rural | -0.02 (-0.07 - 0.04) | 0.02 (-0.06 - 0.10) | NA |
| Year2020 × Rural | -0.04 (-0.12 - 0.04) | 0.11 (-0.003 - 0.23) | NA |
| Year2021 × Rural | 0.12 (0.03 - 0.20)^c^ | 0.18 (0.07 - 0.29)^c^ | NA |
| Year2019 × > 10% below FPL | NA | -0.01 (-0.06 - 0.03) | -0.006 (-0.05 - 0.04) |
| Year2020 × > 10% below FPL | NA | 0.01 (-0.08 - 0.10) | 0.03 (-0.06 - 0.11) |
| Year2021 × > 10% below FPL | NA | -0.09 (-0.19 - 0.02) | -0.06 (-0.16 - 0.04) |
| State F.E. | Yes | Yes | Yes |
| Observations | 3 072 | 2 416 | 2 400 |
| R^2^ | 0.37 | 0.37 | 0.37 |

Abbreviations: OTP, opioid treatment program; CI, confidence interval; FPL, federal poverty level; F.E.: fixed effects.

Source: Authors’ analysis of National Directory of Drug and Alcohol Abuse Treatment Facilities data (2019-2022).

Notes: This analytic sample included 3 112 county-year observations from 778 counties that experienced changes in access to Medicare-accepting-OTPs. In model 1, 40 observations from 10 counties were excluded due to missing information on racial composition. In Model 2, 696 observations from 174 counties were omitted because of missing information on county poverty rates. In Model 3, 712 observations from 178 counties were excluded due to missing data on both county poverty rate and racial composition.

^a^ Counties with more than 27.02% nonwhite residents in 2018.

^b^ Counties with more than 10% residents living below the federal poverty line in 2018.

^c^ P < .01.

^d^ P < .001.

^e^ P < .05.

Table S11. Sensitivity Analyses for Differential Changes in Service Availability at OTPs (2018-2021) Using an Alternative Model

| **Variable** | **Ongoing**  **buprenorphine**  **(β [95% CI])** | **HIV or AIDS education and support**  **(β [95% CI])** | **Employment**  **services**  **(β [95% CI])** | **Comprehensive mental health assessment**  **(β [95% CI])** |
| --- | --- | --- | --- | --- |
| Year2019 | 0.03 (0.006 - 0.06)^a^ | 0.03 (0.008 - 0.05)^c^ | 0.02 (-0.009 - 0.04) | 0.02 (0.004 - 0.03)^a^ |
| Year2020 | 0.06 (0.04 - 0.09)^b^ | 0.05 (0.02 - 0.07)^b^ | 0.02 (-0.01 - 0.05) | 0.03 (0.01 - 0.05)^c^ |
| Year2021 | 0.07 (0.04 - 0.10)^b^ | 0.06 (0.03 - 0.08)^b^ | 0.04 (0.0002 - 0.07)^a^ | 0.04 (0.02 - 0.07)^c^ |
| Year2019 × Type1 | 0.02 (-0.03 - 0.07) | -0.008 (-0.04 - 0.03) | 0.03 (-0.03 - 0.09) | -0.004 (-0.03 - 0.02) |
| Year2020 × Type1 | 0.03 (-0.04 - 0.09) | -0.001 (-0.05 - 0.05) | 0.06. (-0.006 - 0.13) | 0.01 (-0.03 - 0.05) |
| Year2021 × Type1 | 0.03 (-0.04 - 0.09) | 0.002 (-0.06 - 0.07) | 0.01 (-0.07 - 0.10) | -0.01 (-0.06 - 0.03) |
| Year2019 × Type2 | -0.04 (-0.08 - 0.01) | 0.01 (-0.03 - 0.05) | 0.03 (-0.03 - 0.08) | 0.04 (-0.01 - 0.09) |
| Year2020 × Type2 | -0.05 (-0.10 - -0.0002)^a^ | -0.009 (-0.06 - 0.04) | 0.05 (-0.01 - 0.11) | 0.03 (-0.03 - 0.09) |
| Year2021 × Type2 | -0.04 (-0.09 - 0.02) | 0.03 (-0.03 - 0.09) | 0.06 (-0.01 - 0.14) | 0.04 (-0.03 - 0.10) |
| Facility F.E. | Yes | Yes | Yes | Yes |
| Intercept | Yes | Yes | Yes | Yes |
| Observations | 3 380 | 3 380 | 3 380 | 3 380 |
| R^2^ | 0.83 | 0.80 | 0.84 | 0.89 |

Abbreviations: OTP, opioid treatment program; CI, confidence interval; F.E.: fixed effects.

Source: Authors’ analysis of National Directory of Drug and Alcohol Abuse Treatment Facilities data (2019-2022).

Notes: These analyses assessed differential changes in service availability at type 1 OTPs (i.e., OTPs that never accepted Medicare from 2018 to 2021) and type 2 OTPs (i.e., OTPs that always accepted Medicare from 2018 to 2021), compared to type 0 OTPs (i.e., OTPs that newly accepted Medicare after 2018). The analytic sample is a balanced longitudinal sample including 3 380 observations from 845 OTPs. Of the 868 OTPs in the primary balanced longitudinal sample, 23 type 3 OTPs switched to not accepting Medicare or changed their Medicare acceptance status more than once. The 92 observations from these OTPs were excluded from the analyses.

^a^ P < .05.

^b^ P < .001.

^b^ P < .01
